# Graphene oxide–metal oxide nanocomposites: fabrication, characterization and removal of cationic rhodamine B dye†

**DOI:** 10.1039/c8ra00977e

**Published:** 2018-04-10

**Authors:** Nagi M. El-Shafai, Mohamed E. El-Khouly, Maged El-Kemary, Mohamed S. Ramadan, Mamdouh S. Masoud

**Affiliations:** Department of Chemistry, Faculty of Science, Alexandria University Alexandria Egypt; Institute of Nanoscience and Nanotechnology, Kafrelsheikh University Kafr El-Sheikh 33516 Egypt; Department of Chemistry, Faculty of Science, Kafrelsheikh University Kafr El-Sheikh 33516 Egypt mohamedelkhouly@yahoo.com

## Abstract

The fabrication and characterization of graphene oxide (GO) nanosheets and their reaction with Fe_3_O_4_ and ZrO_2_ metal oxides to form two nanocomposites, namely graphene oxide–iron oxide (GO–Fe_3_O_4_) and graphene oxide–iron oxide–zirconium oxide (GO–Fe_3_O_4_@ZrO_2_), have been examined. The fabricated nanocomposites were examined using different techniques, *e.g.*transmission electron microscopy, X-ray diffraction, zeta potential measurement and Fourier transform infrared spectroscopy. Compared to GO, the newly fabricated GO–Fe_3_O_4_ and GO–Fe_3_O_4_@ZrO_2_ nanocomposites have the advantage of smaller band gaps, which result in increased adsorption capacity and photocatalytic effects. The results also showed the great effect of the examined GO–metal oxide nanocomposites on the decomposition of cationic rhodamine B dye, as indicated by steady-state absorption and fluorescence, time correlated single photon counting and nanosecond laser photolysis techniques. The antibacterial activity of the fabricated GO and GO–metal oxides has been studied against Gram-positive and Gram-negative bacteria.

## Abstract 2

The fabrication and characterization of graphene oxide–iron oxide and graphene oxide–iron oxide–zirconium oxide nanocomposites have been reported. The decomposition of cationic rhodamine B dye by both nanocomposites has been examined.

## Graphical abstract

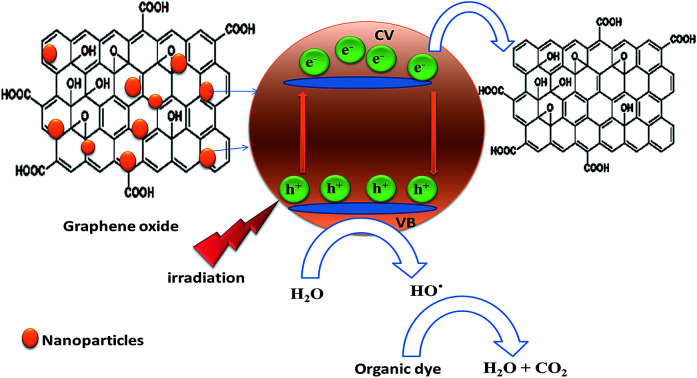

## 1. Introduction

Water pollution has been a vital environmental issue for the last few decades.^1,2^ Industrial organic dyes and heavy metals are considered to be the most important sources of water pollution.^1,2^ For this purpose, membrane technologies based on nanomaterials have been extensively examined for water purification and desalination over the last few decades. Among the utilized nanomaterials in water treatment, graphene oxide (GO), with its fascinating 2D carbon framework with a honeycomb-like structure, has attracted much attention in the last decade for its unique specific surface area, high charge carrier mobility and electron conductivity.^3–5^

Recently, there has been great interest in fabricating and utilizing novel graphene oxide–metal oxide nanocomposites for environmental remediation by the degradation and elimination of toxic organic contaminants and heavy metals, and for antibacterial applications.^6–8^ Compared with graphene oxide, graphene oxide–metal oxide nanocomposites show a unique structural morphology and photochemical properties which render them good candidates for water treatment projects.^9–11^ Among the utilized metal oxides, zero valent iron (ZVI) has been widely used for separating water from harmful heavy metals^12,13^ and organic species.^14–22^ The fabricated graphene oxide–iron oxide nanocomposites showed high efficiency in the removal of tiny concentrations of chromium ions from water and industrial waste water.^23–28^ In addition, the zirconium oxide (ZrO_2_) nanoparticles showed unique electrochemical properties when combined with graphene oxide.^29–36^ Compared with the widely used TiO_2_, zirconium oxide (ZrO_2_) is less expensive and insoluble in water. According to the preparation method, ZrO_2_ exhibited a band gap ranging from 3 to 5 eV. Such a wide band gap renders ZrO_2_ a promising photocatalyst for the production of hydrogen in water decomposition.^37^

Taking these unique properties into consideration, we report herein the fabrication and characterization of GO, graphene oxide–iron oxide (GO–Fe_3_O_4_) and graphene oxide–iron oxide–zirconium oxide (GO–Fe_3_O_4_@ZrO_2_). This combination of graphene oxide with Fe_3_O_4_–ZrO_2_ metal oxide and its application in the degradation of organic species is rare in the literature. Photocatalytic studies of the examined nanocomposites on the degradation of cationic rhodamine B dye (RhB) have been examined in detail using TEM, XRD, FTIR, steady-state absorption and fluorescence and nanosecond laser flash photolysis techniques.

## 2. Experimental section

### 2.1. Chemicals and materials

All of the chemicals and reagents were from Aldrich Chemicals and used without any further purification.

### 2.2. Characterization techniques

UV-vis absorption and fluorescence measurements were taken using a Shimadzu UV-2450 spectrophotometer and a Shimadzu RF-5301PC spectrofluorometer, respectively. Picosecond time-resolved fluorescence lifetimes were recorded on a Fluo300 (PicoQuant, Germany). Lifetimes were evaluated using FluoFit software, which was attached to the equipment. Nanosecond transient absorption studies were recorded using a nanosecond laser flash photolysis technique (LP980, Edinburgh Instruments, UK). The instrument was connected with a tunable laser source (NT342B-10, Ekspla). Fourier transform infrared (FT-IR) spectra were obtained using a JASCO spectrometer 4100, using a KBr pellet technique. The X-ray diffraction (XRD) measurements were conducted using a Shimadzu 6000 model with Cu Kα (*λ* = 1.5418 Å) as the incident radiation. Transmission electron microscopy (TEM) images were taken using a JEOL 2010 microscope operating under a maximum acceleration voltage of 200 kV. Zeta potential results were obtained using a Brookhaven zeta potential/particle size analyzer.

### 2.3. Photocatalytic activity

The photocatalytic activities of GO, GO–Fe_3_O_4_@ZrO_2_ and GO–Fe_3_O_4_ nanocomposites were evaluated for the adsorption of dyes, such as rhodamine B, without light and their efficiency for the degradation of rhodamine B (RhB) dye under visible light irradiation (simulator of sunlight; 150 W Xenon lamp, *λ* > 420 nm). 1 × 10^−4^ M RhB dye and 2 mg nanocomposite were dispersed in 10 ml H_2_O. Measurements were performed every 5 min after exposure to visible light. This experiment was repeated using UV light at 256 nm. OH˙ radicals were generated more during the reaction, which can result in the rapid degradation of RhB dye molecules.^38^ The photo degradation of RhB by graphene oxide–metal oxide nanocomposites was analyzed using steady-state absorbance and fluorescence, time-resolved fluorescence and nanosecond laser photolysis techniques.

### 2.4. Synthesis of nanocomposites based on graphene oxide (GO)

#### 2.4.1 Synthesis of the GO nanostructure

Water dispersions and solid graphite oxide were prepared from natural graphite powder using a modified Hummers and Offeman’s method.^39,40^ In a typical reaction, 8 g graphite flakes (Sigma Aldrich), 8 g NH_4_NO_3_ and 368 ml 98% (w/w) H_2_SO_4_ were mixed under stirring in an ice bath for 1 h. Then, 40 g KMnO_4_ was slowly added to the mixture in the ice bath until the solution became green. The beaker was placed in a water bath at 35 °C and the solution was stirred for about 1 h to form a thick paste. 640 ml high-purity water was then added to the formed paste and stirred at 90 °C for 1 h. The formed solution turned brown. With the slow addition of 48 ml H_2_O_2_ (30%), the color changed from dark brown to yellow. The solid was filtered, washed with 10% HCl aqueous solution (3.2 L) to remove metal ions and washed with water several times. The resulting graphene oxide was dried at 45 °C for 24 h. The crystalline structure of the GO powder was identified using an XRD technique. Refinement was carried out from a starting model based on information given in the Inorganic Crystal Structure Database (ICSD). The morphology of the synthesized GO was examined using transmission electron microscopy (TEM, JEOL 2100) under a maximum acceleration voltage of 200 kV.

#### 2.4.2 Synthesis of the GO–Fe_3_O_4_@ZrO_2_ nanocomposite

0.04 g GO sheets were dispersed in 100 ml water for around 30 minutes. Then, 100 ml of the prepared solution (ZrOCl_2_·8H_2_O, 0.064 M FeCl_2_·4H_2_O and 0.129 M FeCl_3_·6H_2_O) was added. To the resulting mixture, KOH solution in ethanol (1 M, 90 °C) was added dropwise under stirring for 1 hour at 100 °C. At the end, we obtained a black precipitate that was harvested using centrifugation and washed with both water and ethanol. The formed GO–Fe_3_O_4_@ZrO_2_ nanocomposite was dried under vacuum at 45 °C.

#### 2.4.3 Synthesis of the GO–Fe_3_O_4_ nanocomposite

0.04 g GO sheets were dispersed in 100 ml water for 30 minutes using ultrasound and 100 ml aqueous solution (FeCl_2_·4H_2_O and FeCl_3_·6H_2_O) was then added. To this mixture, KOH in ethanol (1 M) was added dropwise under stirring for 1 h at 100 °C. The obtained black precipitate (GO–Fe_3_O_4_) was harvested using centrifugation and washed with water and ethanol. The GO–Fe_3_O_4_ nanocomposite was dried under vacuum at 45 °C.

## 3. Results and discussion

### 3.1. Characterization of GO, GO–Fe_3_O_4_ and GO–Fe_3_O_4_@ZrO_2_ nanocomposites

**Fig. 1:**
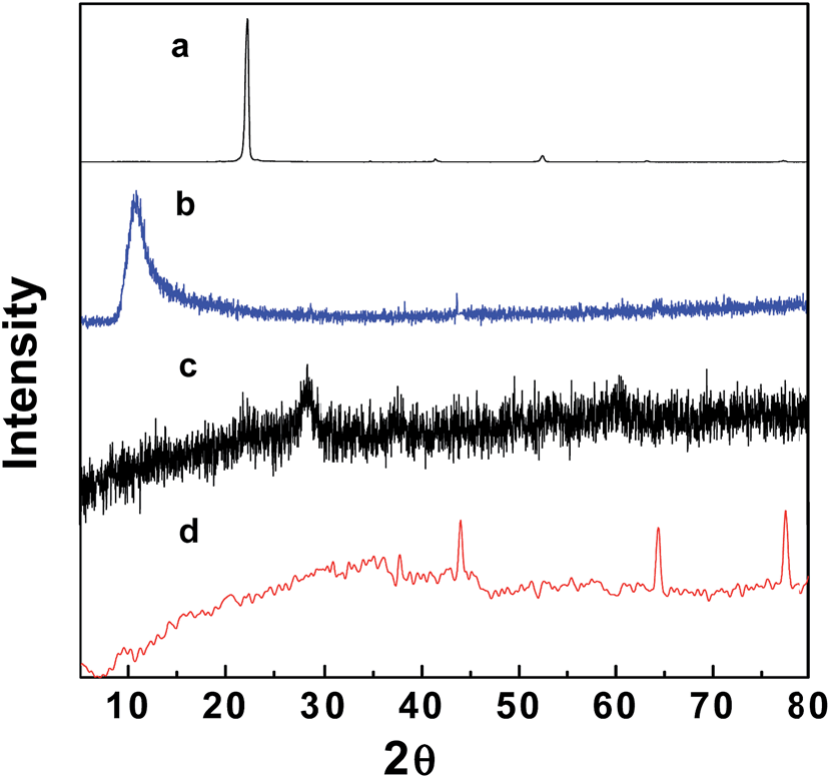
XRD patterns of: (a) graphite, (b) graphene oxide, (c) GO–Fe_3_O_4_ and (d) GO–Fe_3_O_4_@ZrO_2_.

Fig. 1 and S1† show the XRD patterns of the fabricated GO–Fe_3_O_4_ and GO–Fe_3_O_4_@ZrO_2_ nanocomposites, in addition to those of pure graphite powder, GO, Fe_3_O_4_ and ZrO_2_. The XRD pattern of GO exhibited a diffraction peak at 10.9°,^41,42^ which is significantly larger than that found in the XRD pattern of pure graphite (26.0°). This can be rationalized by the presence of oxygenated functional groups on the carbon sheets of GO.^43,44^ XRD analysis of GO–Fe_3_O_4_ showed diffraction peaks at 29.6°, 35.3°, 43.5°, 56.5° and 63.5°,^45^ while GO–Fe_3_O_4_@ZrO_2_ showed peaks at 44.0°, 64.2° and 77.3°, as observed from the database, and a single phase with a monoclinic structure was formed. The main crystallite sizes of the GO and metal oxide nanocomposites were calculated based on the Debye–Scherrer formula (eqn (1)).^46^

1 *D* = *Kλ*/*β* cos *θ*

where *K* is a constant representing the shape factor (∼0.9), *λ* is the wavelength of the X-ray source (1.5405 Å), *β* is the full width at half maximum of the diffraction peak and *θ* is the angular position of the peak. The average crystallite sizes of GO–Fe_3_O_4_ and GO–Fe_3_O_4_@ZrO_2_ were determined to be 8 and 10 nm, respectively.

**Fig. 2:**
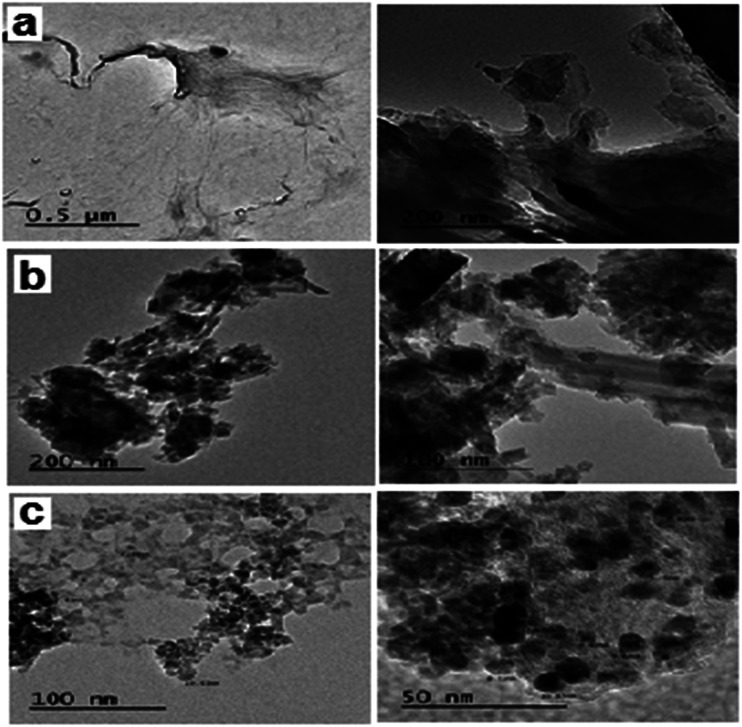
TEM images of: (a) GO, (b) GO–Fe_3_O_4_@ZrO_2_ and (c) GO–Fe_3_O_4_.

Fig. 2 shows the TEM images of the fabricated GO, GO–Fe_3_O_4_ and GO–Fe_3_O_4_@ZrO_2_ nanocomposites with different magnifications. From the images, GO appeared as nano-sheets, while GO–Fe_3_O_4_ and GO–Fe_3_O_4_@ZrO_2_ appeared as nano-spherical shapes. The samples were analyzed using EDX with uniform particle morphology (Fig. S2†). The average size of the observed metal oxides on the surface of graphene oxide was ∼8 to 10 nm, which is in good agreement with that observed using XRD.

The absorption spectra of fabricated GO–Fe_3_O_4_ and GO–Fe_3_O_4_@ZrO_2_ were recorded in water, as shown in Fig S3.† The absorption spectra exhibited an absorption peak with a maximum at ∼228 nm, which was attributed to the π → π* transitions of the aromatic C=C bonds.^47,48^ The absorption bands at 390 and 360 nm correspond to GO–Fe_3_O_4_@ZrO_2_ and GO–Fe_3_O_4_, respectively. The band gap values of the nanocomposites were determined using eqn (2):^49,50^

2 *αhυ* = *A*(*hυ* − *E*_g_)^*n*^

where *α* is the absorption coefficient, *υ* is the frequency of light, *h* is Planck’s constant, *hυ* is the photon energy, *A* is a proportionality constant, *E*_g_ is the band gap and *n* = 1/2 for the direct transitions.^51^ From the plot of (*αhυ*)^2^*versus hυ* (the insets of Fig. S3†), the band gaps for GO, GO–Fe_3_O_4_@ZrO_2_ and GO–Fe_3_O_4_ were found to be 4.00, 3.20 and 3.66 eV, respectively.

**Fig. 3:**
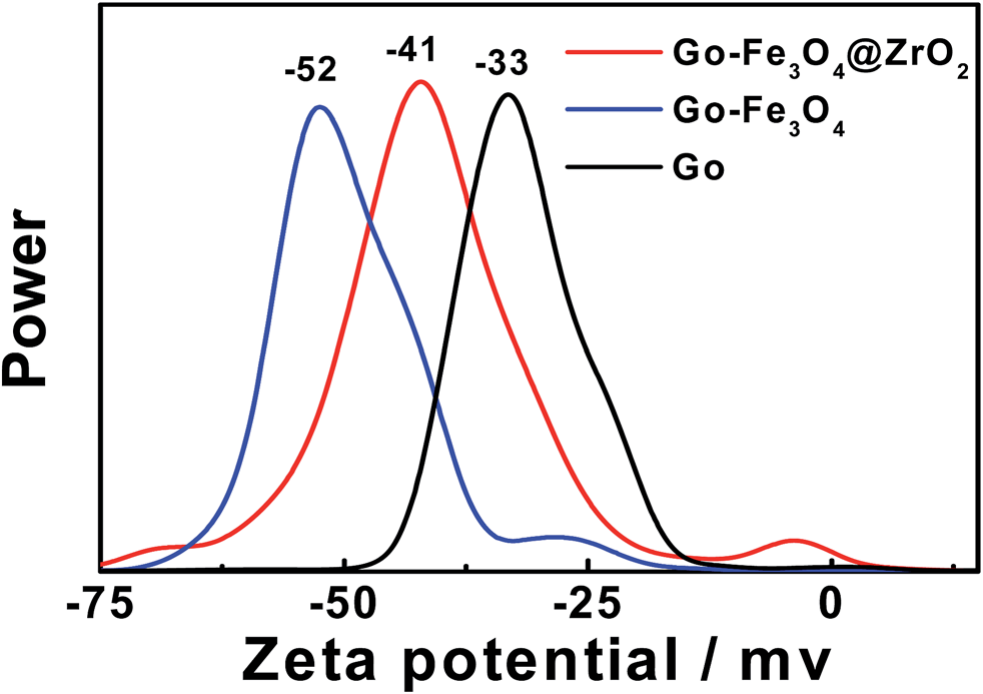
The zeta potentials of GO, GO–Fe_3_O_4_@ZrO_2_ and GO–Fe_3_O_4_ in water.

A zeta potential technique has been used to predict the long term stability of the nanoparticles in solution and to understand the state of the nanoparticle surface. As shown in Fig. 3, the spectra show negatively charged particles for GO (−33), GO–Fe_3_O_4_@ZrO_2_ (−41) and GO–Fe_3_O_4_ (−52). These negative values are related to the stability of the colloidal dispersions in water.

**Fig. 4:**
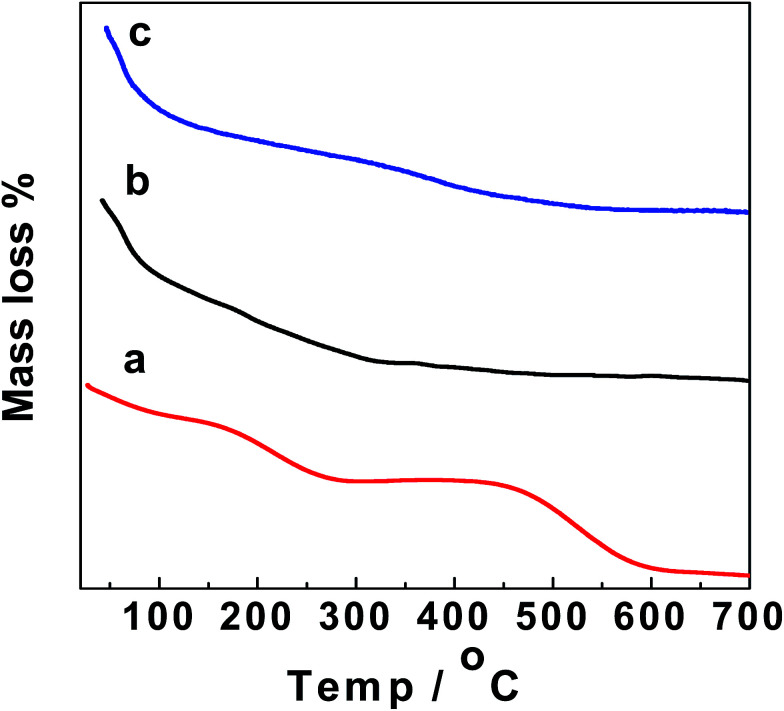
TGA of: (a) GO, (b) GO–Fe_3_O_4_@ZrO_2_ and (c) GO–Fe_3_O_4_.

Fig. 4 shows a thermogravimetric analysis (TGA) diagram for GO, GO–Fe_3_O_4_@ZrO_2_ and GO–Fe_3_O_4_ in the range of 25–700 °C. As seen from the TGA steps, the diagram shows that the decomposition steps of GO with changing temperature match the decomposition steps of GO–Fe_3_O_4_@ZrO_2_ and GO–Fe_3_O_4_, suggesting the successful loading of the metal oxides over the GO surface.

**Fig. 5:**
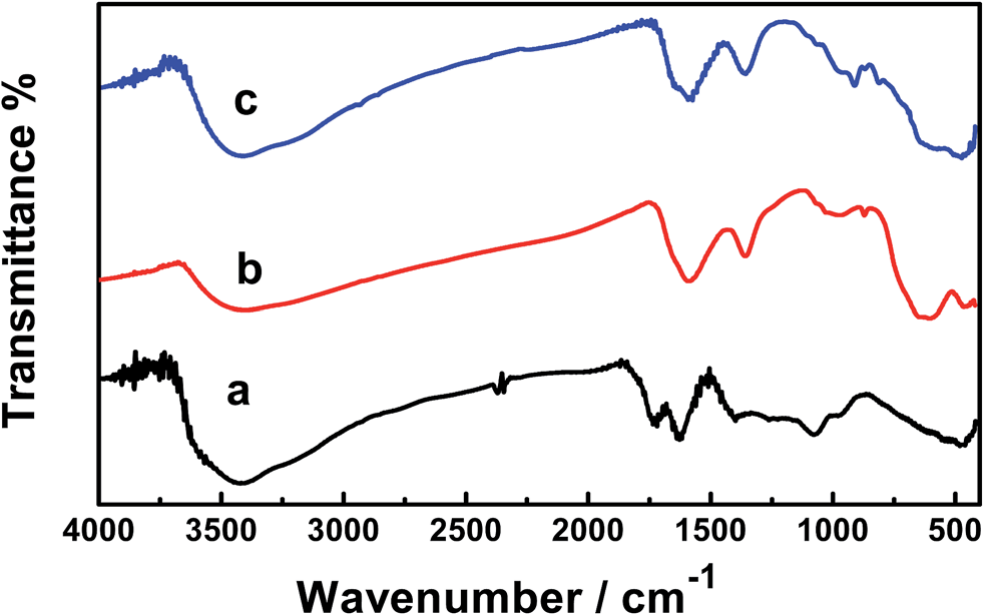
FT-IR spectra of: (a) GO, (b) GO–Fe_3_O_4_ and (c) GO–Fe_3_O_4_@ZrO_2_.

Fig. 5 shows the FTIR spectra of the GO, GO–Fe_3_O_4_–ZrO_2_ and GO–Fe_3_O_4_ nanocomposites. The peaks observed at 508 cm^−1^ correspond to the characteristic stretching vibrations of the C–O bond in GO nanoparticles.^52^ The characteristic peaks of GO vibration were recorded at 984 to 506 cm^−1^. The broad absorption band observed at ∼3455 cm^−1^ corresponds to the stretching vibration of the O–H band of physically absorbed water.^53^ The recorded peaks at 573 cm^−1^ (for GO–Fe_3_O_4_@ZrO_2_) and 587 cm^−1^ (for GO–Fe_3_O_4_) correspond to the characteristic vibrations of the M–O bond.^54^

### 3.2. Photocatalytic study of rhodamine B

#### 3.2.1 Photocatalytic study of RhB under UV irradiation

**Fig. 6:**
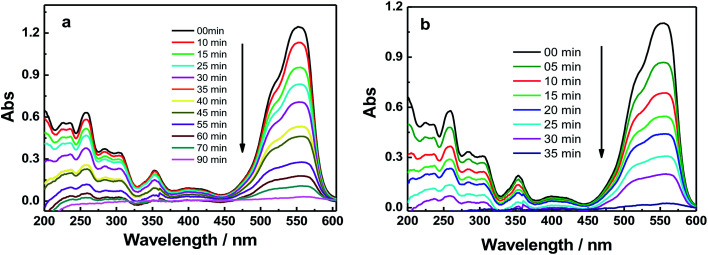
UV-absorbance spectra of RhB in the presence of: (a) GO and (b) GO–Fe_3_O_4_@ZrO_2_ in water at the indicated time intervals under UV irradiation at 256 nm.

Fig. 6 and S4† show the absorption spectra of the photocatalytic degradation of RhB using GO, GO–Fe_3_O_4_@ZrO_2_ and GO–Fe_3_O_4_ nanocomposites under UV irradiation at 256 nm. As shown, the characteristic absorption band of RhB dye was recorded at 554 nm. With increasing irradiation time, the absorption band of RhB at 554 nm is greatly decreased and red-shifted in the presence of GO nanocomposites. In a control experiment, the absorption band of RhB at 554 nm showed no significant changes under irradiation in the absence of GO nanocomposites (Fig. S5†).

The observed rate constants for the photocatalytic degradation of RhB with GO nanocomposites were determined using eqn (3):

3 ln(*C*/*C*_o_)= −*k*_obs_*t*

where *C*_o_ (mg l^−1^) is the initial dye concentration and *k*_obs_ depends on the initial dye concentration (*C*_o_).^55,56^ From the linear plot of ln(*C*/*C*_o_) with irradiation time, the degradation rates (*k*) of RhB in the presence of GO, GO–Fe_3_O_4_@ZrO_2_ and GO–Fe_3_O_4_ were determined to be 0.0479, 0.0997 and 0.05328 min^−1^, respectively. This finding indicates a higher rate constant in the case of the GO–Fe_3_O_4_@ZrO_2_ nanocomposite compared to that of GO.

**Fig. 7:**
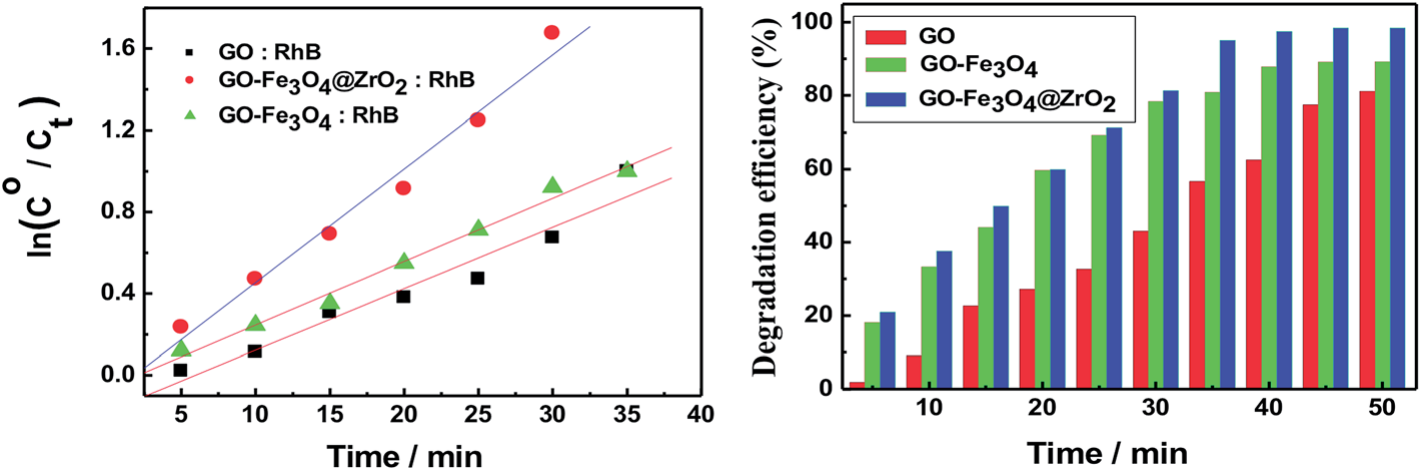
(Left) Pseudo-first-order plots for the photodegradation of RhB with GO, GO–Fe_3_O_4_@ZrO_2_ and GO–Fe_3_O_4_, with increasing irradiation time. (Right) The photo-degradation efficiency (%) of RhB with GO, GO–Fe_3_O_4_@ZrO_2_ and GO–Fe_3_O_4_, with changing irradiation time (*λ* = 256 nm).

**Scheme 1:**
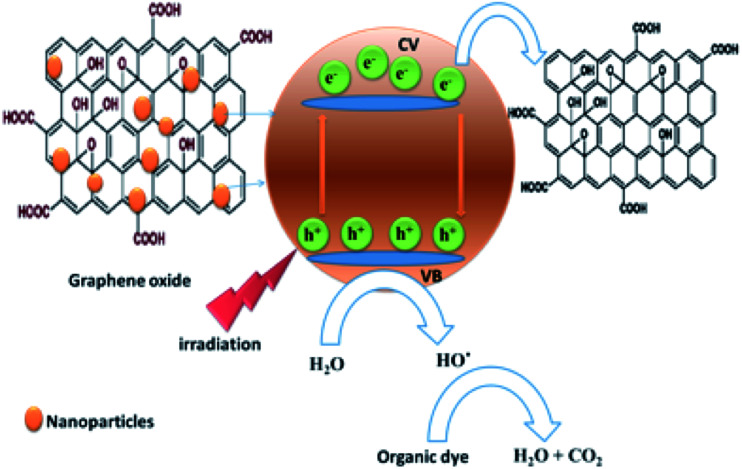
The proposed mechanism for the photodegradation of cationic dye by graphene oxide–metal oxide nanocomposites.

The efficiency of the photocatalytic degradation process was determined using eqn (4):

4 *D* (%) = [*A*(RhB)_o_ − *A*(RhB)_*t*_]/*A*(RhB)_o_

where *A*(RhB)_o_ and *A*(RhB)_*t*_ are the absorbance changes of RhB at 554 nm with time, in the dark and under light irradiation, respectively.^57,58^Fig. 7 illustrates that 80% of the RhB dye was degraded in the presence of GO within around 90 minutes. This percentage was increased to 90% and 98% of RhB in the presence of Fe_3_O_4_ and Fe_3_O_4_@ZrO_2_, respectively, suggesting the significant effect of the examined metal oxides (Fe_3_O_4_ and Fe_3_O_4_@ZrO_2_) in increasing the photocatalytic degradation of RhB/GO composites. This high efficiency for RhB degradation by GO–Fe_3_O_4_@ZrO_2_ (98%) is found to be considerably higher compared to that reported for TiO_2_–rGO (81%).^59^ The proposed mechanism for the photodegradation of cationic RhB dye using GO–metal oxide nanocomposites is shown in Scheme 1,^60–63^ where the surface of GO has the ability to receive electrons from the high conduction band (CB) of ZrO_2_, which reacts with oxygen to produce superoxide anion radicals (O_2_˙^−^) and OH˙ radicals, leading to the rapid oxidation of the organic molecules.

#### 3.2.2 Photocatalytic study of RhB under a sunlight simulator

**Fig. 8:**
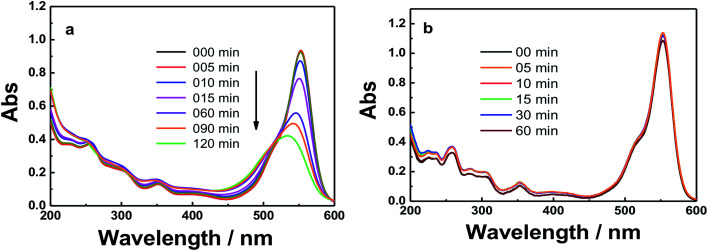
Absorbance spectra of RhB dye in the presence of: (a) GO and (b) GO–Fe_3_O_4_@ZrO_2_ at the indicated time intervals under irradiation.

The photocatalytic degradation of RhB was examined with GO, GO–Fe_3_O_4_@ZrO_2_ and GO–Fe_3_O_4_ nanocomposites under visible light irradiation (sunlight simulator, UXL-151D-O, Xe 150 W, *λ* > 420 nm). As shown in Fig. 8 and S6,† the results showed no photocatalytic activity from the GO–Fe_3_O_4_@ZrO_2_ and GO–Fe_3_O_4_ nanocomposites toward RhB. On the other hand, GO showed a low efficiency that may be explained by the adsorption process of the dye over the GO surface.

### 3.3. Adsorption process of RhB on the surface of GO nanocomposites

**Fig. 9:**
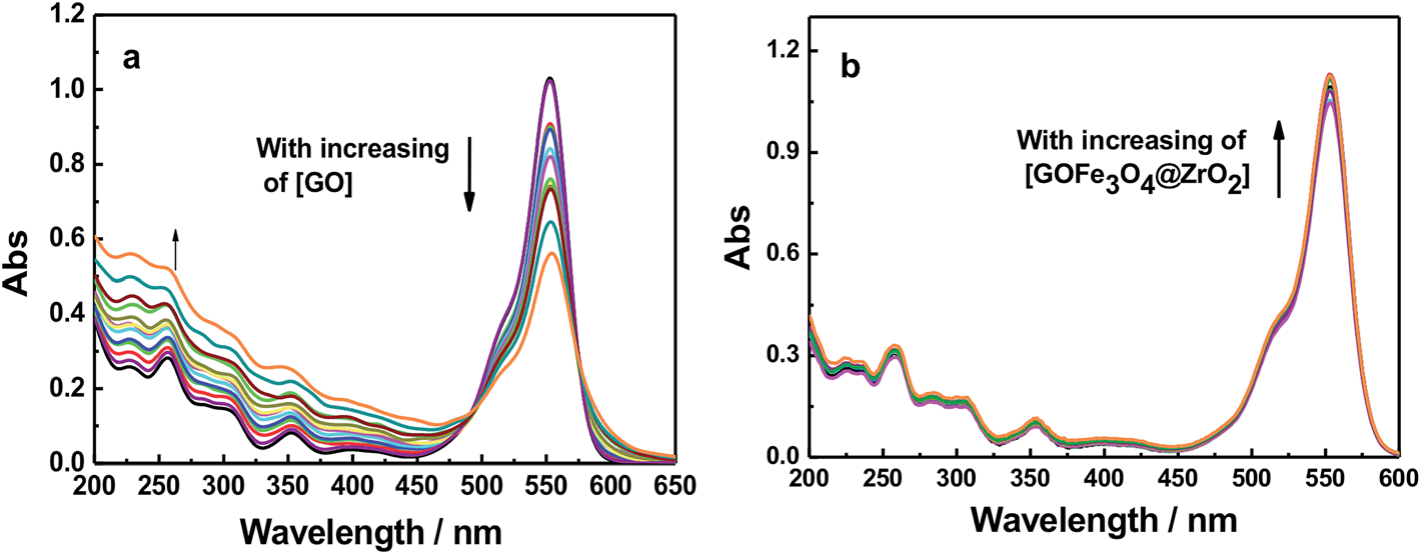
Absorption spectra of RhB (1.7 × 10^−5^ M) with different concentrations of (a) GO and (b) GO–Fe_3_O_4_@ZrO_2_ in water.

**Fig. 10:**
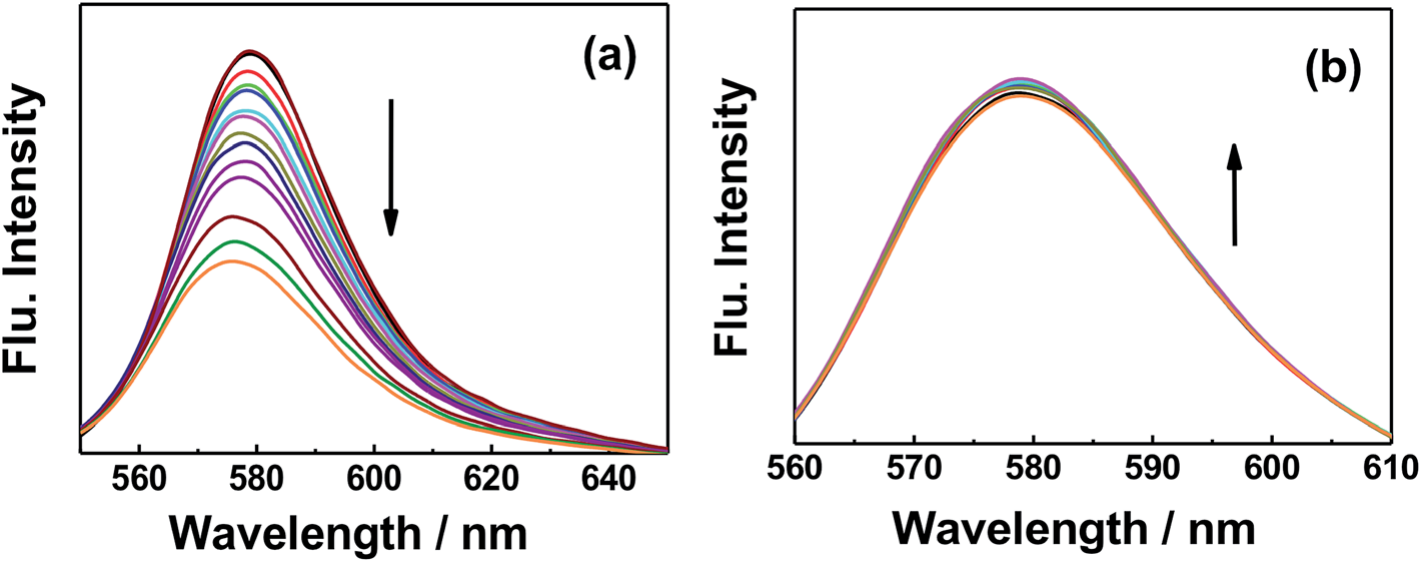
Fluorescence changes of RhB (1.7 × 10^−5^ M) with the addition of different concentrations of (a) GO and (b) GO–Fe_3_O_4_@ZrO_2_ in water.

Dye molecules were entrapped on the surface of GO and GO-nanocomposites in aqueous solution. From Fig. 9 and S7,† one can see that the absorption band of RhB at 550 nm decreased gradually with increasing amounts of GO (0.2 g l^−1^). For GO–Fe_3_O_4_ and GO–Fe_3_O_4_@ZrO_2_, different features were observed where the absorption band of RhB was considerably increased with increasing amounts of GO–Fe_3_O_4_ and GO–Fe_3_O_4_@ZrO_2_. The fluorescence measurements showed the same trend as observed for the absorption studies. As seen in Fig. 10 and S8,† the fluorescence maximum band of RhB at 579 nm was significantly decreased in the presence of GO, but not in the presence of GO–Fe_3_O_4_ or GO–Fe_3_O_4_@ZrO_2_. The adsorption process could be explained by the dye binding with GO through hydrogen bonding, and electrostatic interactions.^64,65^

### 3.4. Laser studies of the photodegradation process of RhB by GO composites

**Fig. 11:**
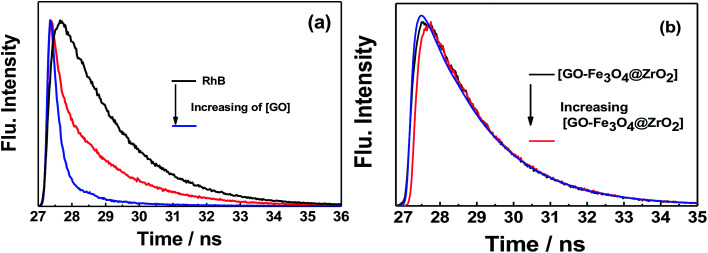
Fluorescence decay profiles of the singlet-excited state of RhB in the presence of (a) GO and (b) GO–Fe_3_O_4_@ZrO_2_ in water; *λ*_ex_ = 420 nm; *λ*_em_ = 580 nm.

Fluorescence lifetime measurements showed the same trend as observed for the fluorescence measurements (Fig. 11 and S9†). Upon exciting RhB with 470 nm laser light, the fluorescence decay–time profile of the singlet-excited state of RhB (^1^RhB*) decayed with a monoexponential decay, from which the fluorescence lifetime of ^1^RhB* was determined to be 1.7 ns. With increasing amounts of GO, the substantial quenching of the fluorescence lifetime was considerable and the decay could be fitted satisfactorily to a biexponential decay. The fast decaying component had a lifetime of 120 ps (58%), while the slow decaying component had a lifetime of 1.9 ns (42%). The lifetime of the slow decaying component is close to that of the free RhB. Based on the change in the recorded fluorescence lifetimes of RhB in the absence and presence of GO, the rate and efficiency of the quenching process were determined to be 7.74 × 10^9^ s^−1^ and 93%, respectively.^66–68^ For GO–Fe_3_O_4_ and GO–Fe_3_O_4_@ZrO_2_, it was observed that the fluorescence lifetime of ^1^RhB* was kept almost the same with increasing concentrations of both GO–Fe_3_O_4_ and GO–Fe_3_O_4_@ZrO_2_. These measurements are in good agreement with the steady-state fluorescence measurements. These measurements suggest a higher adsorption of RhB over the surface of GO, but not GO–Fe_3_O_4_ and GO–Fe_3_O_4_@ZrO_2_.

**Fig. 12:**
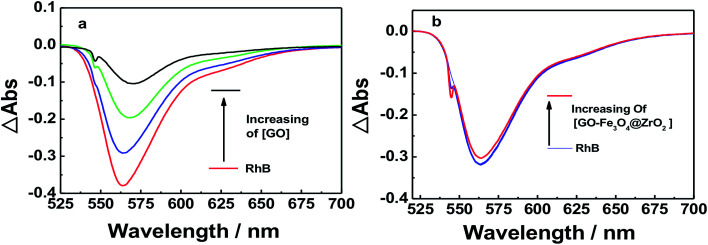
Nanosecond transient absorption spectra of RhB dye in the presence of (a) GO and (b) GO–Fe_3_O_4_@ZrO_2_ in an oxygen-free water solution; *λ*_ex_ = 550 nm.

Nanosecond transient absorption spectroscopy was used to obtain further insight into the excited state interactions of RhB with GO, GO–Fe_3_O_4_@ZrO_2_ and GO–Fe_3_O_4_, to corroborate the observed interaction by both steady-state and time-resolved fluorescence techniques. To achieve this, RhB dye was probed with excitation at *λ* = 550 nm in an oxygen-free water solution. The nanosecond transient absorption spectrum of RhB in water was dominated by pronounced bleaching between 540 and 600 nm, which was due to the depletion of the singlet ground state (Fig. 12 and S10†). In the case of RhB–GO, it was observed that the singlet state of RhB recovered quickly with increasing amounts of GO, confirming the quenching of the singlet state of RhB by the GO. In the case of RhB with GO–Fe_3_O_4_@ZrO_2_, the intensity of the ground state bleaching remained almost unchanged with increasing amounts of GO–Fe_3_O_4_@ZrO_2_, suggesting that there was no interaction between RhB and Fe_3_O_4_@ZrO_2_.

### 3.5. Antibacterial activity of GO and nanocomposites

**Fig. 13:**
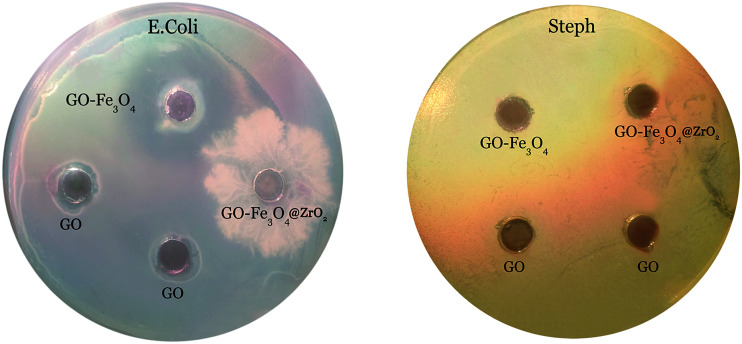
Zone of inhibition tests against *E. Coli* and *Steph* strains in the presence of GO, GO–Fe_3_O_4_@ZrO_2_ and GO–Fe_3_O_4_, at a concentration of 0.5 mg ml^−1^.

Antibacterial activity was tested against Gram-positive and Gram-negative bacteria using BHI agar plates and the agar diffusion method. The GO, GO–Fe_3_O_4_@ZrO_2_ and GO–Fe_3_O_4_ samples were evaluated. The resulting antibacterial effect could be rationalized by the diffusion of GO, Fe_3_O_4_@ZrO_2_ and GO–Fe_3_O_4_ over the agar surface, preventing bacterial growth in the specific area occupied by the nanocomposite. As seen from Fig. 13, we observed only a small zone of inhibition around GO, indicating limited bacterial toxicity against *E. coli*.^69^ In contrast, the GO–Fe_3_O_4_ sample showed a significant inhibitory effect against *E. coli*. The presence of clear zones on the BHI agar surface proves that the GO–Fe_3_O_4_ composite was able to inhibit the growth of *E. coli*, whereas no antibacterial activity was detected for raw GO and GO–Fe_3_O_4_@ZrO_2_ against *E. coli*. The GO, Fe_3_O_4_@ZrO_2_ and GO–Fe_3_O_4_ samples showed no antibacterial activity against *Steph*. The experiment was conducted to characterize bacterial killing with concentration (0.5 mg ml^−1^) and the cellular viability was measured after 24 h exposure time.

## 4. Conclusion

Novel nanocomposites of graphene oxide with iron oxide (GO–Fe_3_O_4_) and iron oxide–zirconium oxide (GO–Fe_3_O_4_@ZrO_2_) were fabricated and characterized using XRD, TGA, FTIR, and TEM techniques. From the optical absorption measurements, the energy band gap values were found to be 4.00, 3.66, and 3.20 eV for GO, GO–Fe_3_O_4_ and GO–Fe_3_O_4_@ZrO_2_, respectively. All of the steady-state absorption and fluorescence, time-resolved fluorescence and nanosecond transient absorption spectroscopy results confirmed that RhB is efficiently adsorbed over the surface of graphene oxide (∼93%). Different features were observed in the presence of metal oxides (Fe_3_O_4_ and Fe_3_O_4_@ZrO_2_) over the surface of graphene oxide. GO and Fe_3_O_4_@ZrO_2_ had a small zone of inhibition against *E. coli* and in contrast, the GO–Fe_3_O_4_ sample showed a significant inhibitory effect against *E. coli*. GO, GO–Fe_3_O_4_@ZrO_2_ and GO–Fe_3_O_4_ showed no antibacterial activity against *Steph*.

## Conflicts of interest

The authors declare no conflict of interest.

## Supplementary Material

RA-008-C8RA00977E-s001
