# Supplementary material for: Graphene oxide–metal oxide nanocomposites: fabrication, characterization and removal of cationic rhodamine B dye
Source: RSC Adv. 2018 Apr 10;8(24):13323–32. doi: 10.1039/c8ra00977e (PMC9079739; doi:10.1039/c8ra00977e)
Supplement: RA-008-C8RA00977E-s001 [file RA-008-C8RA00977E-s001.pdf]

Electronic Supplementary Material (ESI) for RSC Advances.
This journal is © The Royal Society of Chemistry 2018

Supporting Information`s for RSC Advances

Graphene Oxide – Metal Oxide Nanocomposites: Fabrication, Characterization and Removal of Cationic Rhodamine-B Dye

Nagi M. El-Shafai,[ab] Mohamed E. El-Khouly,*[c] Maged El-Kemary,*[bc] Mohamed S. Ramadan,[a] Mamdouh S. Masoud*[a]

[a] *Department of Chemistry, Faculty of Science, Alexandria University, Alexandria, Egypt*

[b] *Institute of Nanoscience and Nanotechnology, Kafrelsheikh University, Kafr El-Sheikh 33516, Egypt*

[c] *Department of Chemistry, Faculty of Science, Kafrelsheikh University, Kafr El-Sheikh 33516, Egypt*

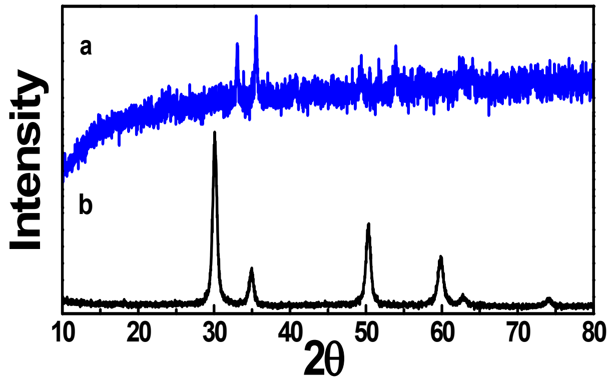

Fig. S1 XRD patterns of: (a) Fe_3O_4,NPs (b) ZrO_2 NP

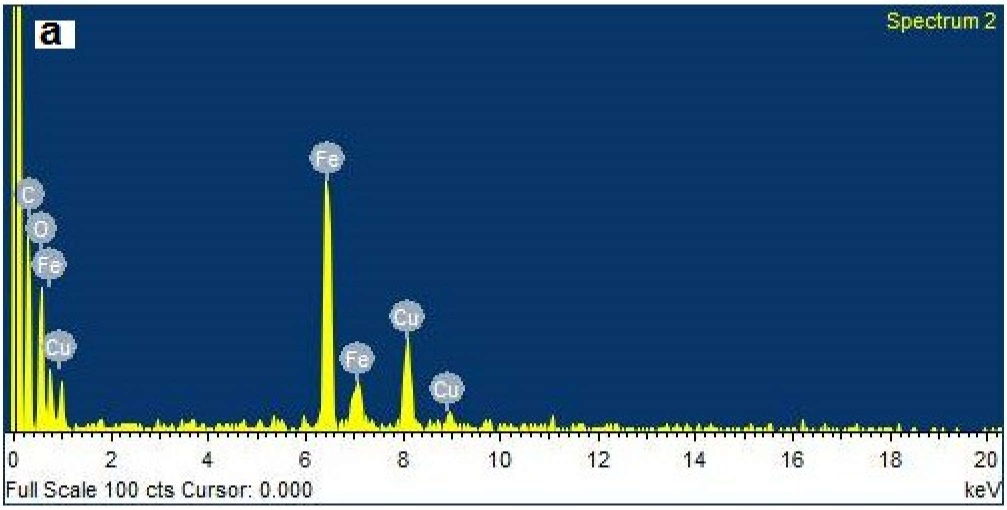

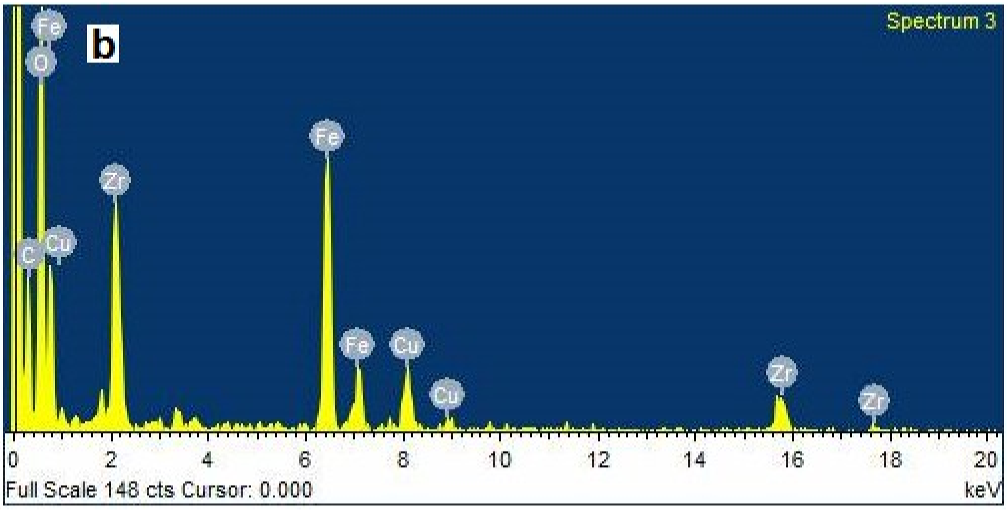

Fig. S2 EDX analysis of: (a) GO-Fe_3O_4, and (b) GO-Fe_3O_4@ZrO_2.

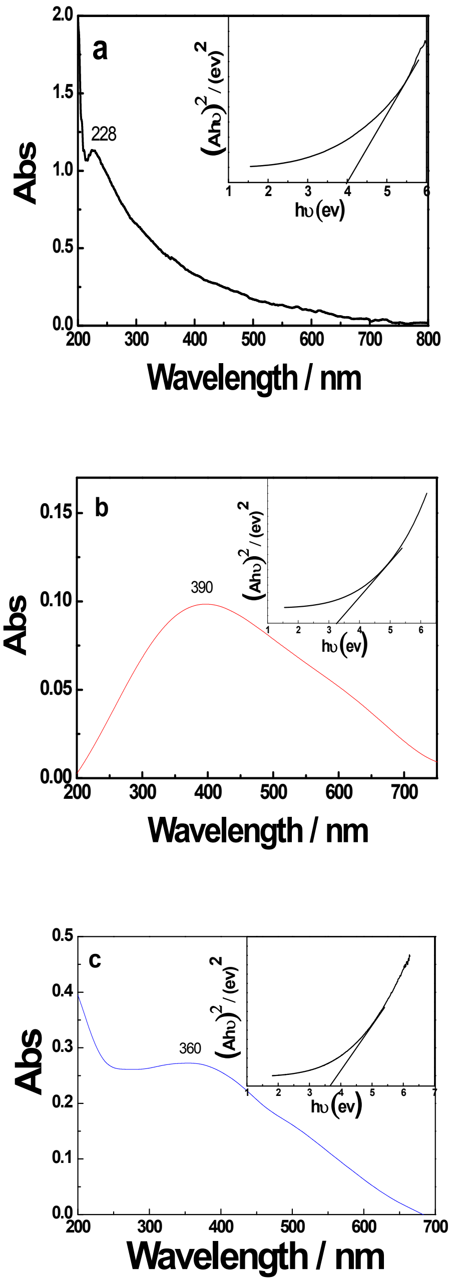

Fig. S3 UV-visible absorbance spectra of: (a) GO, (b) GO-Fe_3O_4@ZrO_2, and (c) and GO-Fe_3O_4 in water.

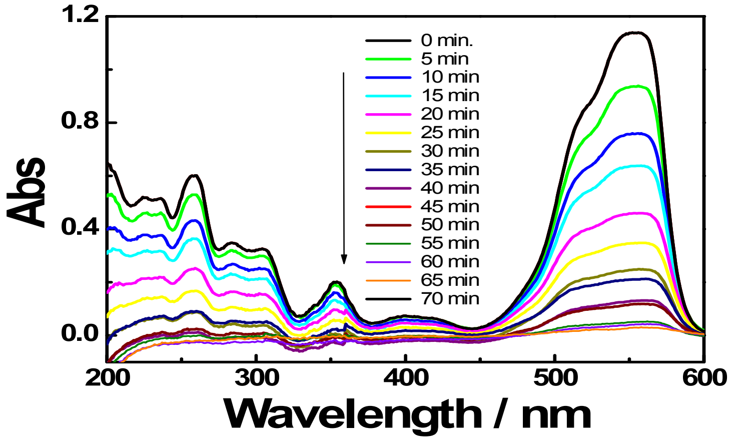

Fig. S4 UV-absorbance spectra of RhB in the presence of GO-Fe_3O_4 in water at the indicated time intervals under UV irradiation at 256 nm.

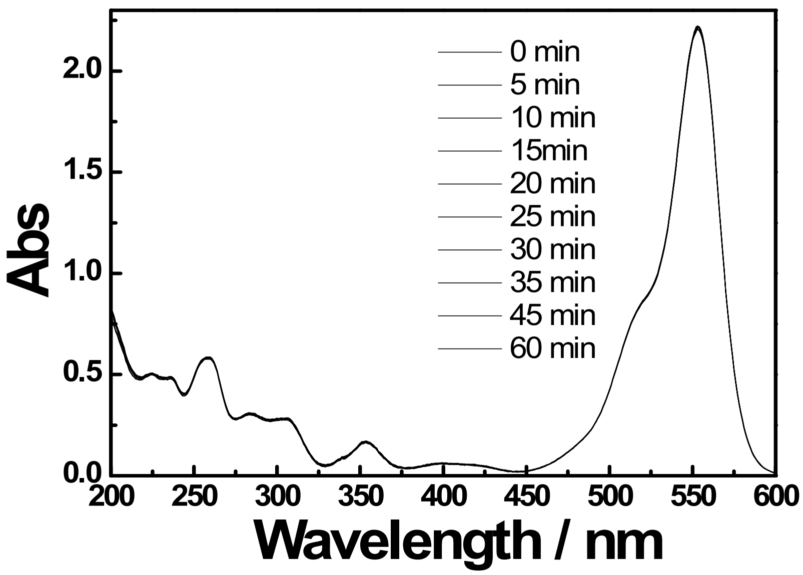

Fig. S5. UV absorbance spectrum of RhB in water with different time under radiation.

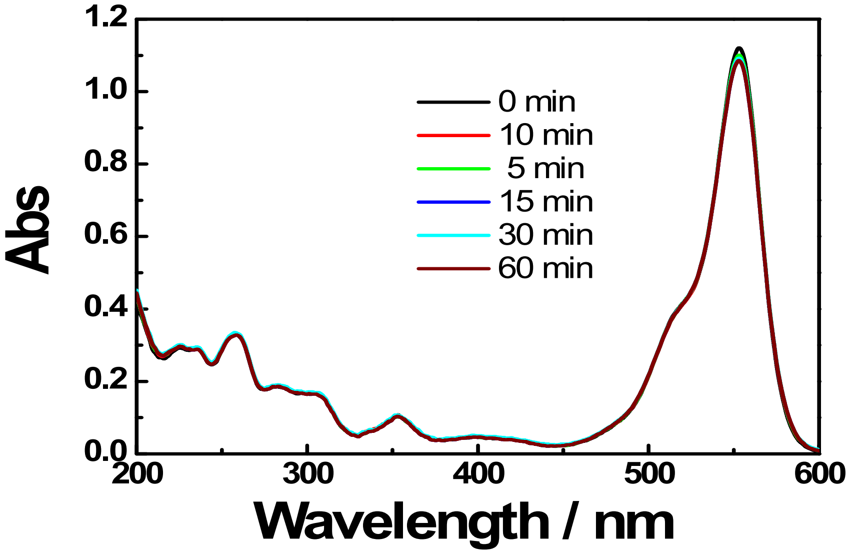

Fig. S6. UV- absorbance spectra of RhB in the presence of GO-Fe_3O_4 in water with different time under simulator of sun light.

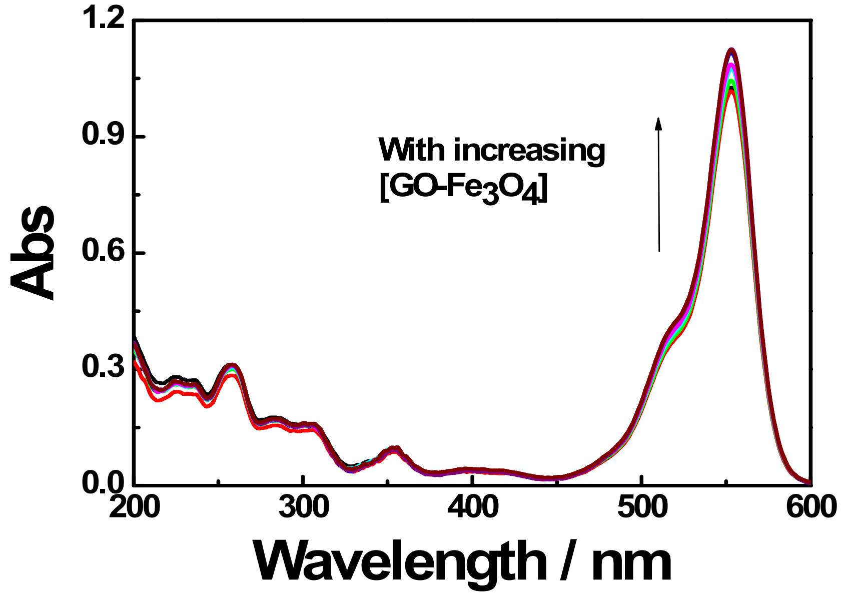

Fig. S7. UV-visible absorption spectra of RhB (1.7×10^{-5} M) with different concentrations of GO-Fe_3O_4 in water.

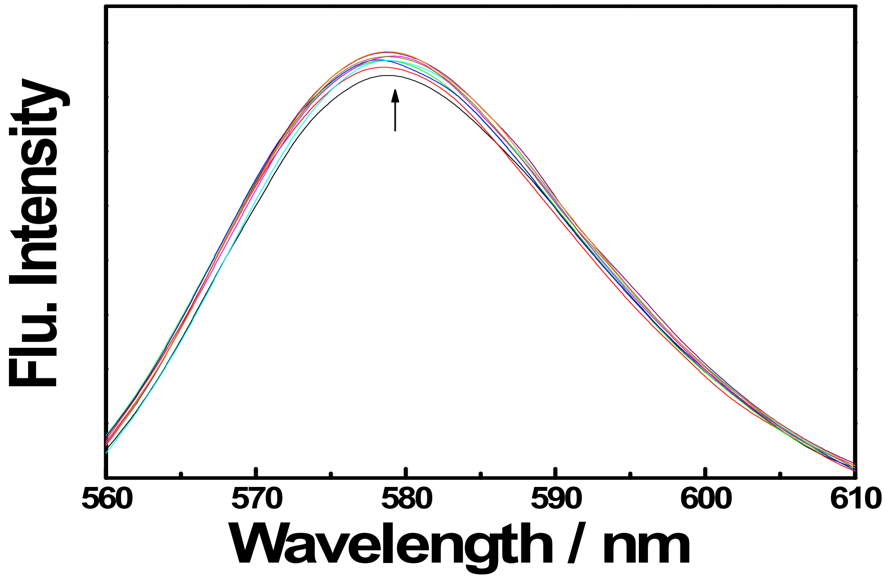

Fig. S8. Fluorescence spectra of RhB (1.7×10^{-5} M) with different concentrations of $GO\text{-}Fe_3O_4$ without radiation in water.

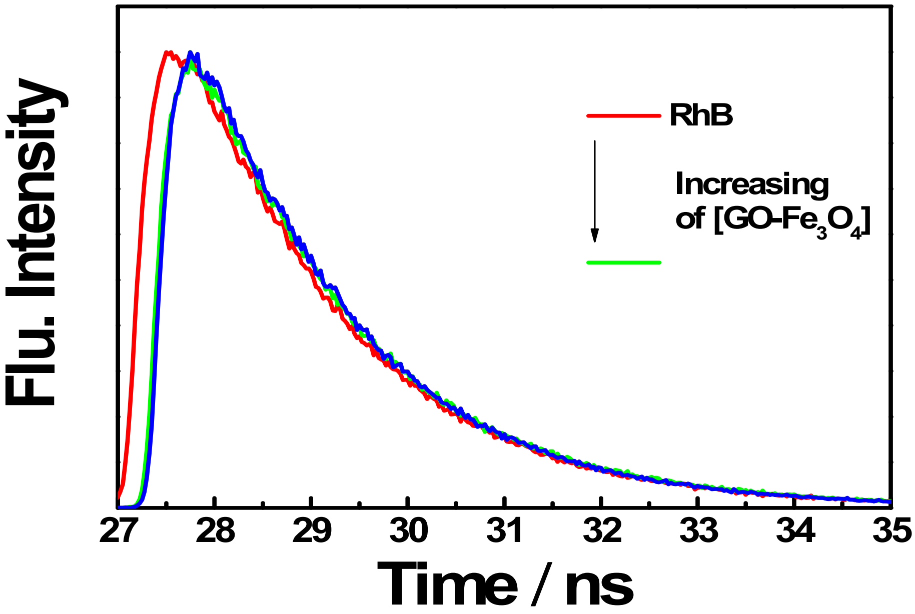

Fig. S9. Fluorescence decay profiles of the singlet-excited state of RhB in the presence of $GO\text{-}Fe_3O_4$ in water; $\lambda_{ex} = 420$ nm; $\lambda_{em} = 580$ nm.

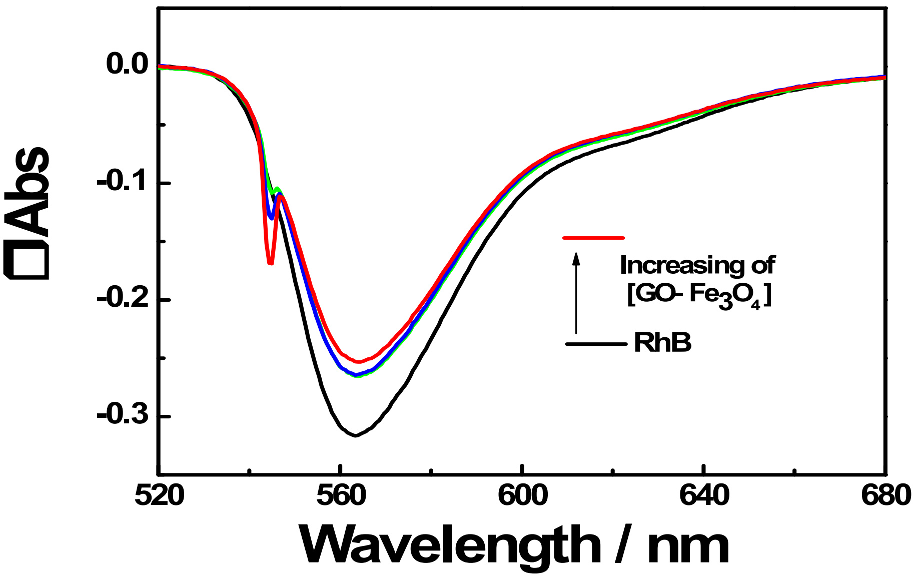

Fig. S10 Nanosecond transient absorption spectra of RhB in the presence of GO-Fe_3O_4 in oxygen-free water solutions; λ_{ex} = 550 nm.
